# Supplementary material for: Bone corticalization requires local SOCS3 activity and is promoted by androgen action via interleukin-6
Source: Nat Commun. 2017 Oct 9;8:806. doi: 10.1038/s41467-017-00920-x (PMC5634449; doi:10.1038/s41467-017-00920-x)
Supplement: Supplementary file 1 — Supplementary Information [file 41467_2017_920_MOESM1_ESM.pdf]

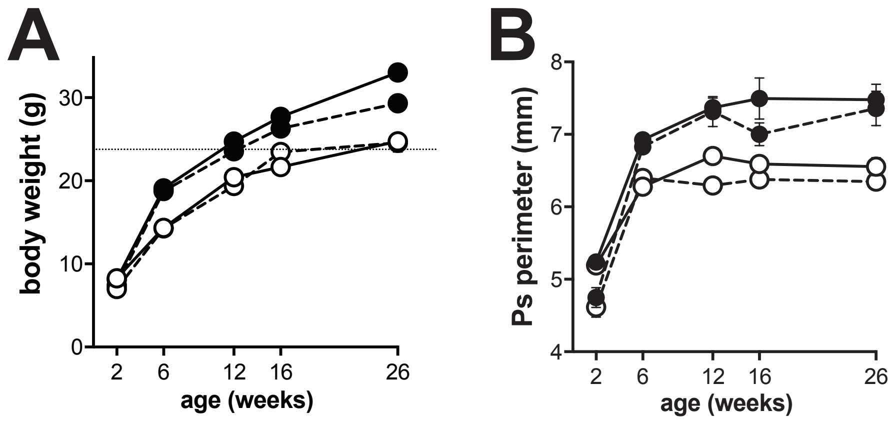

Supplementary Figure 1: Body weight and femoral periosteal circumference in *Dmp1Cre.Socs3$^{f/f}$* and *Dmp1Cre* control mice: A: Body weights (A) and femoral periosteal (Ps) perimeter (B) of 2, 6, 12, 16 and 26 week old male (filled circles) and female (open circles) *Dmp1Cre.Socs3$^{f/f}$* (dashed lines – f/f) and *Dmp1Cre* (solid lines – w/w) mice. Values are mean+SEM; numbers are as follows in the order female w/w, female f/f, male w/w, male f/f: 2 weeks (7, 8, 8, 7); 6 weeks (9, 11, 12, 10); 12 weeks (11, 10, 10, 9); 16 weeks (9, 8, 11, 7); 26 weeks (9, 9, 9, 10).

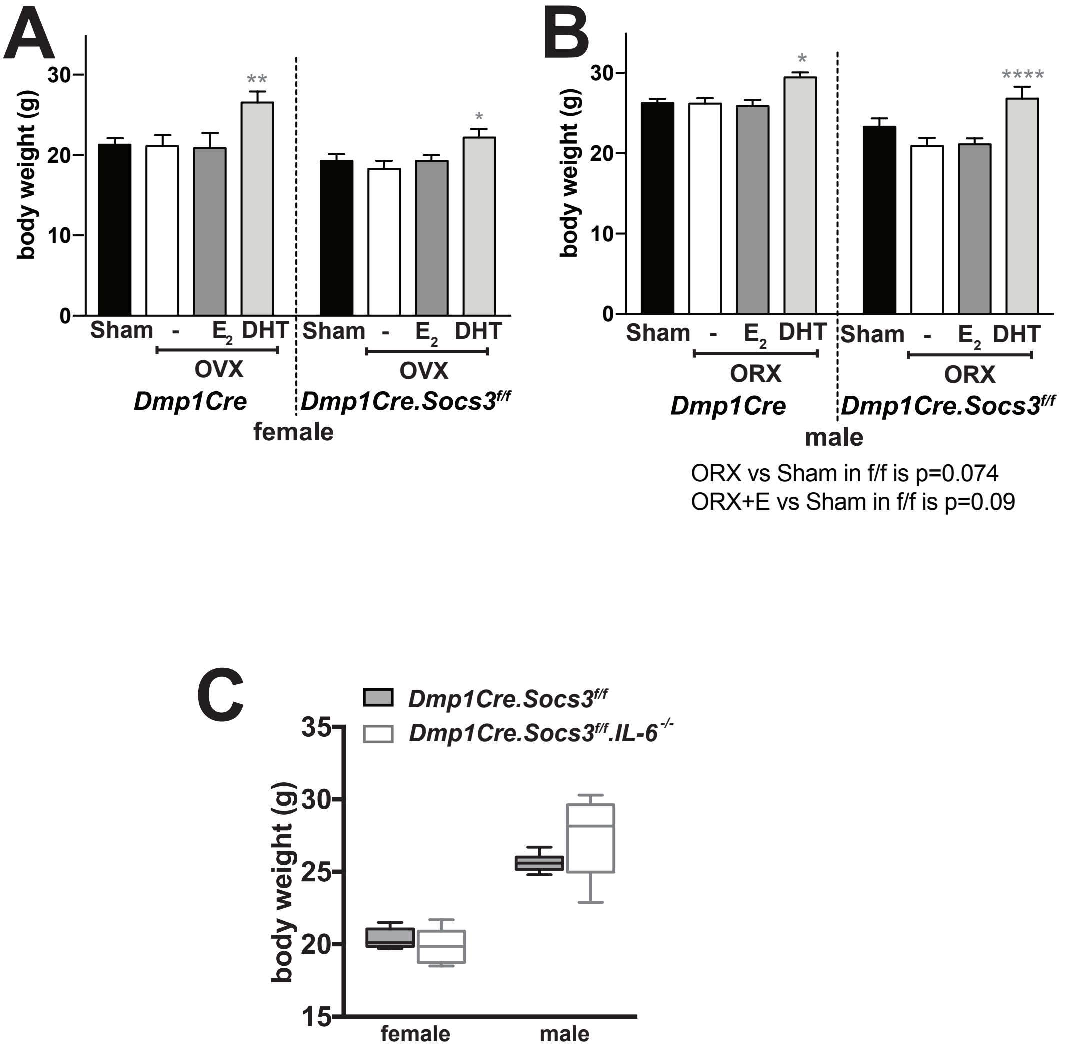

Supplementary Figure 2: Body weights of female (**A**) and male (**B**) *Dmp1Cre* and *Dmp1Cre.Socs3$^{f/f}$* mice subjected to gonadectomy and steroid treatments outlined in Figure 5A. Values are mean+SEM, n=6/group. *, $p<0.05$; ****, $p<0.0001$ vs sham operated mice of the same genotype. **C:** Body weights of 12 week old female and male *Dmp1Cre.Socs3$^{f/f}$* and *Dmp1Cre.Socs3$^{f/f}$.IL-6$^{-/-}$* littermates. n=7-8 per group.
